# Capecitabine-induced Subacute Cutaneous Lupus Erythematosus^☆^^☆☆^

**DOI:** 10.1016/j.abd.2019.09.004

**Published:** 2019-09-30

**Authors:** Aroni Rocha, Hiram Larangeira de Almeida, Gustavo Zerwes, Umberto Lopes de Oliveira Filho

**Affiliations:** a Department of Dermatology, Universidade Federal de Pelotas, Pelotas, RS, Brazil; b CERON Clinic, Pelotas, RS, Brazil; c Department of Reumatology, Universidade Federal de Pelotas, Pelotas, RS, Brazil

Dear Editor,

Capecitabine is a chemotherapeutic fluoropyrimidine that acts as a prodrug, being metabolized into 5-fluorouracil through an enzymatic cascade, and is used for treatment of solid colorectal, gastric, and breast tumors. Its use has become popular in recent years due to its similar efficacy when compared to 5-fluorouracil, with a more tolerable toxicity profile and the convenience of oral administration.^1^ Systemic toxicity attributed to capecitabine includes gastrointestinal manifestations such as nausea, vomiting, and diarrhea. Known dermatologic side effects are hand-foot syndrome (described as painful erythema, edema, and palmoplantar dysesthesia), pyogenic granuloma, vitiligo, onycholysis, and xerosis cutis.^2^

A 53-year-old woman with a history of depression, dyslipidemia, and osteoporosis, was diagnosed, in April 2012, with invasive ductal carcinoma, molecular subtype luminal B. At the time she was treated with a mastectomy of the left breast and axillary node clearance, radiotherapy, and adjuvant chemotherapy with 5-fluorouracil, epirubicin, and cyclophosphamide. In July 2017, the patient presented a pleural effusion, whose immunohistochemical study showed disease relapse. She then started treatment with capecitabine in October 2017, with 14 days on and seven days off the drug at a dosage of 2000 mg/m^2^/daily. She developed a rash on her face, neck, and scalp after six weeks of capecitabine use. The patient did not present fever, arthralgia, or myalgia. Physical examination showed erythematous, scaly patches along the V-line of the upper chest and interscapular region; there was also diffuse erythema in the androgenetic alopecia region, which pointed to a photosensitive component (Fig. 1A and B). Parallel to this, the patient also developed painful erythema on her hands and feet, consistent with hand-foot syndrome, a well-known side effect of some chemotherapeutic agents such as capecitabine (Fig. 1C and D).^3^ Laboratory exams were within normal range or negative: blood count, urinalysis, ANA, anti-double-stranded DNA antibodies, anti-histone antibodies, and anti-La/SSB antibodies. Anti-Ro/SSA antibodies were positive, 68 IU/mL (normal range until 10 IU/mL).

**Figure 1:**
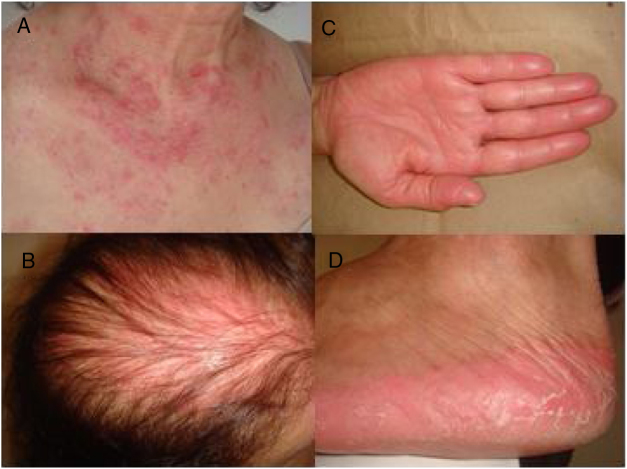
(A) Erythematous lesions in the presternal region; (B) Erythema in the androgenetic alopecia region; (C and D) Hand-foot syndrome with erythema and desquamation.

Histological examination showed apoptotic keratinocytes and pigment incontinence, with a discrete lymphocytic infiltrate (Fig. 2).

**Figure 2:**
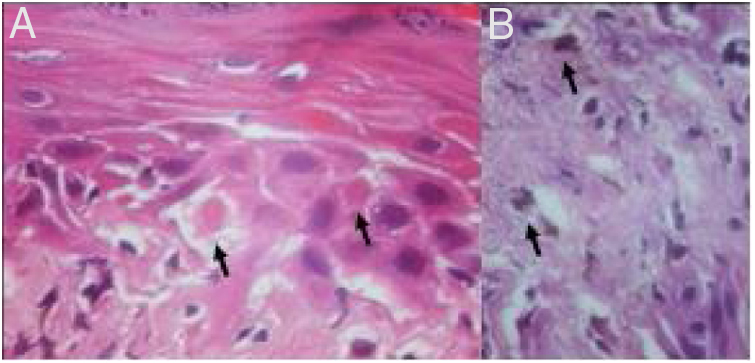
(A) Necrotic keratinocytes (arrows; hematoxylin and eosin, ×400). (B) Pigment incontinence (arrows; hematoxylin and eosin ×400).

Clinical, histological, and laboratorial findings were compatible with the diagnosis of subacute cutaneous lupus erythematosus (SCLE), and capecitabine was defined as the offending drug.

Topical treatment with betamethasone and sun-protective measures were taken and, after capecitabine cessation, the patient showed great improvement of the skin lesions.

SCLE is characterized by erythemato-squamous and annular lesions, with remarkable photosensitivity, typically associated with elevated serum levels of anti-Ro antibodies. It can be categorized as drug-induced or idiopathic, both forms being indistinguishable in clinical, serological, and histological aspects.

Since the first report of drug-induced SCLE, associated with the use of hydrochlorothiazide, a growing number of medications have been described as culprits for the syndrome.^4^ Medications classically associated with drug-induced SCLE, such as calcium channel blockers, diuretics, and antifungals, e.g., terbinafine, have given way to proton-pump inhibitors and chemotherapeutic agents as the leading causative agents.^5^

A PubMed and MEDLINE review showed only eight cases of capecitabine-induced SCLE in the literature, and there have been no cases reported in Brazil up to now. This may be caused by an under-reported number of cases and/or may be attributed to our limited experience with the agent, which was only approved by the Brazilian Health Surveillance Agency (Agência Nacional de Vigilância Sanitária [ANVISA]), the country's organ responsible for drug regulation, in 2015, for the treatment of colorectal, stomach, and breast cancers.

Although the pathogenesis behind capecitabine-induced SCLE remains unknown, its growing use in different types of cancer proves necessary the report of dermatological manifestations of the drug. The recognition of this side effect by dermatologists is vital, so that drug-induced SCLE may be included early in the differential diagnosis of patients who are using capecitabine and present skin lesions.

## Financial support

None declared.

## Author's contributions

**Aroni Rocha:** Approval of the final version of the manuscript; conception and planning of the study; elaboration and writing of the manuscript; critical review of the literature; critical review of the manuscript.

**Hiram Larangeira de Almeida Jr.:** Approval of the final version of the manuscript; conception and planning of the study; elaboration and writing of the manuscript; intellectual participation in propaedeutic and/or therapeutic conduct of the cases studied; critical review of the literature; critical review of the manuscript.

**Gustavo Zerwes:** Approval of the final version of the manuscript; conception and planning of the study; elaboration and writing of the manuscript; effective participation in research orientation; intellectual participation in propaedeutic and/or therapeutic conduct of the cases studied; critical review of the literature; critical review of the manuscript.

**Umberto Lopes de Oliveira Filho:** Approval of the final version of the manuscript; conception and planning of the study; elaboration and writing of the manuscript; effective participation in research orientation; intellectual participation in propaedeutic and/or therapeutic conduct of the cases studied; critical review of the literature; critical review of the manuscript.

## Conflicts of interest

None declared.
